# The Plasma Membrane: A Platform for Intra- and Intercellular Redox Signaling

**DOI:** 10.3390/antiox7110168

**Published:** 2018-11-20

**Authors:** Daniela E. Nordzieke, Iria Medraño-Fernandez

**Affiliations:** 1 Institute of Microbiology and Genetics, Department of Genetics of Eukaryotic Microorganisms, Georg August University Göttingen, Grisebachstr. 8, D-37077 Göttingen, Germany; 2 Protein Transport and Secretion Unit, Division of Genetics and Cell Biology, Istituto di Ricovero e Cura a Carattere Scientifico (IRCCS) Ospedale San Raffaele, Università Vita-Salute San Raffaele, 20132 Milan, Italy

**Keywords:** plasma membrane, redox signaling, lipid rafts, NADPH oxidase, aquaporin, redoxosome, inflammation, inflammatory bowel disease, neurodegenerative disorders

## Abstract

Membranes are of outmost importance to allow for specific signal transduction due to their ability to localize, amplify, and direct signals. However, due to the double-edged nature of reactive oxygen species (ROS)—toxic at high concentrations but essential signal molecules—subcellular localization of ROS-producing systems to the plasma membrane has been traditionally regarded as a protective strategy to defend cells from unwanted side-effects. Nevertheless, specialized regions, such as lipid rafts and caveolae, house and regulate the activated/inhibited states of important ROS-producing systems and concentrate redox targets, demonstrating that plasma membrane functions may go beyond acting as a securing lipid barrier. This is nicely evinced by nicotinamide adenine dinucleotide phosphate (NADPH)-oxidases (NOX), enzymes whose primary function is to generate ROS and which have been shown to reside in specific lipid compartments. In addition, membrane-inserted bidirectional H_2_O_2_-transporters modulate their conductance precisely during the passage of the molecules through the lipid bilayer, ensuring time-scaled delivery of the signal. This review aims to summarize current evidence supporting the role of the plasma membrane as an organizing center that serves as a platform for redox signal transmission, particularly NOX-driven, providing specificity at the same time that limits undesirable oxidative damage in case of malfunction. As an example of malfunction, we explore several pathological situations in which an inflammatory component is present, such as inflammatory bowel disease and neurodegenerative disorders, to illustrate how dysregulation of plasma-membrane-localized redox signaling impacts normal cell physiology.

## 1. Introduction

When looking to the world surrounding us, it becomes pretty clear that animals and plants are adapted to the particular conditions of the habitats in which they live. Thus, Emperor penguins have four layers of scale-like feathers that isolate them from the cold Antarctic wind, and the Saguaro cactus’s spines point down to conduct into its folds and its base the rare rain that falls in the Sonoran Desert. These adaptations, developed over billions of years and collectively defined as evolution, are preceded by parallel slow changes at the cellular level. However, the extracellular environment is not static through time. Composed by multiple elements and varying even from minute to minute, cells are continuously exposed to an almost infinite combination of environmental states. To adapt to these changes, cells are forced to communicate, implying transmission of messages in the appropriate time-frame to mount an adequate cellular response.

Many of the molecules that are involved in cellular communication never enter the target cell. Instead, these first messengers bind to specific receptors on cell surfaces, triggering a rapid increase in the intracellular levels of a so-called second messenger [1]. This second messenger is normally a small non-protein molecule that readily diffuses inside the cell. Within the cell, it further regulates the activity of multiple signaling proteins, thereby expanding transmission of information downstream through receptor activation in a non-linear way. Amplification, diversification, and distribution of the original signal to all relevant subcellular compartments are thus achieved, ensuring a proper on-time response of the cell as a whole to each stimulus. In addition, the levels of a second messenger can be influenced by multiple independent upstream inputs at the same time, allowing for a more precise modulation of the outcome of a signal. Therefore, cellular response will not only depend on the specific cell stage in which a signal arrives to the plasma membrane, but also on the amplitude, kinetics, and spatial distribution of the second messenger involved.

To grant control of the velocity, duration, and fidelity of transmission, second messengers are characterized by four basic aspects: (i) they are either enzymatically generated or released in a regulated manner into the cytosol through channels; (ii) they are either enzymatically degraded or have their basal levels restored by the action of pumps or through diffusion and reaction with their targets; (iii) their intracellular levels rise and fall within a short time, creating gradients from their origin that determine their effectivity; and (iv) they are specific in action. These features have been defined after decades of intense research on molecules with a widely recognized function as second messengers, such as cyclic adenosine monophosphate (cAMP) [2], diacylglycerol (DAG) [3], nitric oxide (NO) [4], and Ca^2+^ [5], which represents the prototypic second messenger in living cells. Remarkably, some other molecules, despite the substantial body of evidence that, through the years, has linked them with signal transduction [6], have become accepted as second messengers only recently [7,8]. As one of them, reactive oxygen species (ROS) have been traditionally considered as unavoidable toxic wastes that result from metabolic activity, or as the noxious payback for a life developed under aerobic conditions. Indeed, the collective term ROS has too often been used with laxity to group together all of the molecular intermediates derived from the sequential reduction of molecular oxygen (O_2_), even though they present notable differences in terms of stability and reactivity.

## 2. ROS: Signals, Second Messengers, or Simply Foes?

It is generally accepted that the generation of ROS by non-enzymatic mechanisms is a collateral result of ATP synthesis in mitochondria, with production ratios that depend on the cellular metabolic rate and the availability of the initial substrate, O_2_ [9,10]. However, ROS are also generated on purpose in living cells: to date, 31 ROS-generating protein systems have been described in different locations [11], potentially allowing for enzymatic control of ROS production in response to a stimulus. Importantly, in most cases, a cellular membrane (either the plasma membrane or an organelle membrane) separates these enzymes from their putative targets [12].

**Figure 1:**
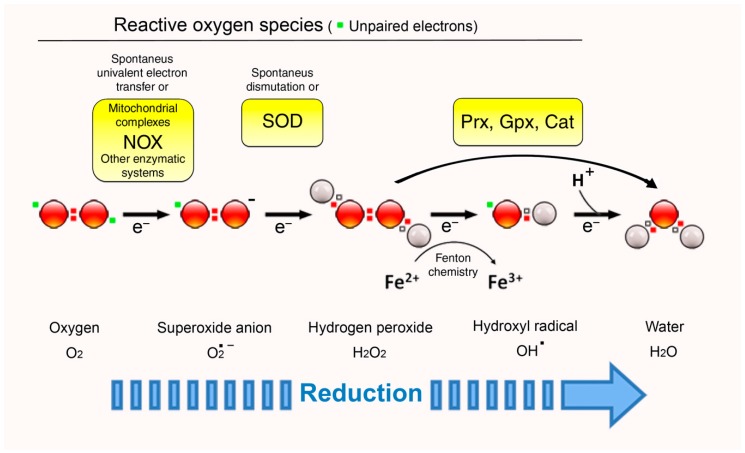
Major producing and removal pathways for reactive oxygen species (ROS). The sequential steps of the univalent reduction of molecular oxygen (O_2_) to water (H_2_O) leading to the generation of several ROS intermediates are shown. Diverse redox enzymatic systems, mainly mitochondrial respiration complexes and membrane-residing NADPH oxidases (NOXes), can convert O_2_ into superoxide (O_2_^•−^). Superoxide dismutases (SOD) catalyze the dismutation of superoxide (O_2_^•−^) into H_2_O_2_ and O_2_. H_2_O_2_ can be reduced directly to water by peroxiredoxins (Prx), glutathione peroxidases (GPX), or catalases (CAT). Alternatively, hydroxyl radicals (OH^•^) are generated from H_2_O_2_ in the presence of reduced transition metals, such as Fe^2+^ (Fenton reaction). Red squares and green squares represent paired and unpaired electrons, respectively, in the oxygen atom. White squares represent the electron provided by hydrogen atoms.

O_2_ in its ground-state is a bi-radical, meaning that it contains two unpaired electrons with parallel spins in its outer valence shell. The unusual electron configuration precludes its direct reaction with many molecules. This includes divalent reductants, implying that the most common mechanism of O_2_ reduction involves the transfer of a single electron (monovalent reduction). The resulting molecules can be either free radicals (containing an unpaired electron in its outer orbit), including a superoxide anion (O_2_^•−^) and a hydroxyl radical (OH^•^), or non-radical oxidants, such as hydrogen peroxide (H_2_O_2_) (Figure 1). These molecules differ in their reactivity, target specificity, half-life, and lipid solubility, and thus are more or less suitable for signaling proposes.

The highly instable and reactive superoxide anion (O_2_^•−^) is the most proximal product of the one-electron reduction of O_2_. It is produced mainly by flavoproteins, such as NADH dehydrogenase in mitochondria [9], NADPH oxidases (NOX) [13], or electron carriers, such as CoQH2 [10,14]. Typical substrates of O_2_^•−^ are iron–sulfur [Fe-S] clusters, whose activation state is frequently regulated by O_2_^•−^-mediated oxidation [15,16]. Independently of [Fe-S]-containing proteins, O_2_^•−^ oxidative activity has been implicated in cellular processes such as autophagy [17] and epigenetic control of gene expression [18]. The question of whether O_2_^•−^ is a relevant signal depends mainly on the scavenging enzyme superoxide dismutase (SOD) [19], as the rate constant for the reaction with SODs is very rapid (2 × 10^9^ M^−1^ s^−1^) and the cytosolic concentration of these enzymes greatly exceeds the steady-state concentration of their substrate [20]. In other words, O_2_^•−^ would only be able to operate as a signaling molecule within a very short distance from its site of generation to avoid dismutation by SODs. Notably, the persistence of a particular redox molecule is intimately linked to the redox environment in which it is produced [21], and it would be feasible that O_2_^•−^ becomes a much more relevant signal when the cellular steady state shifts to a more oxidizing profile [22] in which SODs could be product-inactivated [23].

The product of a spontaneous or SOD-catalyzed O_2_^•−^ conversion is hydrogen peroxide (H_2_O_2_) [24]. The role of this oxidant in signal transduction is nowadays widely accepted, since its chemistry fully adapts to the characteristics that qualify a molecule as a second messenger. First of all, all electrons in H_2_O_2_ are paired (Figure 1), entailing that it is uncharged and relatively unreactive at physiological pH, a fact that redounds on a higher lifetime and stability. Second, H_2_O_2_ can be produced by several enzymatic systems, mostly through univalent reduction of O_2_^•−^ but also directly [11], and is degraded by devoted protein scavengers (catalase, peroxiredoxins, glutathione peroxidases) [25]. Finally, additional strategies, such as compartmentalization of H_2_O_2_ production and regulated distribution using dedicated membrane channels, are employed to preserve homeostatic control of its levels [26]. In a similar way to intracellular Ca^2+^ storage and regulated release, this organizational scheme may contribute to achieve spatiotemporal specificity, allowing for the formation of steep gradients that differentially activate redox-sensitive pathways.

Notwithstanding, H_2_O_2_ can be further reduced to the hydroxyl radical (OH^•^) in the presence of reduced transition metals, such as iron and copper (Fenton Reaction). This radical is highly unstable and quite unselective in oxidation of target molecules and cannot, like O_2_^•−^ and H_2_O_2_, be eliminated by an enzymatic reaction [27]. Therefore, its disposal is mainly the result of its reaction with other macromolecules that are situated in the immediate environment. Analogously to O_2_^•−^, the reactivity of OH^•^ is not a total impediment to its function as a signal in cells: it is conceivable that, under the extreme oxidative conditions in which OH^•^ generation is favored, its reactive nature is exploited to promote a particular cell response, even to activate cell death mechanisms. In that case, OH^•^ may be considered both a signal and an executioner. If this turns out to be true, the lack of specificity brought about by the fast reaction of OH^•^ might be by-passed by strategical positioning of particular targets in close proximity to its sites of production. Along these lines, several studies have related OH^•^ action with specific functions in plants [28,29] and with differentiation of some human cell lines in vitro [30,31]. Likewise, it has been hypothesized that OH^•^-mediated crosslinking is the basis of the supramolecular organization of cell structures, such as the plasma membrane [32].

## 3. Signal Thiol Oxidations Mediated by Hydrogen Peroxide

Over the last decade, the number of reported biological events in which ligand–receptor interaction induces H_2_O_2_-dependent responses has grown exponentially. Accountable for this are at least two of its chemical features: on the one hand, H_2_O_2_ is a strong two-electron oxidant, but on the other it requires high activation energy to start the oxidation of targets [25]. Therefore, this ROS is considered a poor random reactant in vivo, displaying high selectivity on its reactions [33]. Indeed, H_2_O_2_-derived signaling impacts mainly metalloproteins bearing transition metal centers or thiols in specific cysteine or selenocysteine residues [34,35,36], thereby altering their activity and the outcome of the corresponding cellular pathways. Whether a cysteine suits this modification strongly depends on the localization of the residue in the protein, its exposition to the surrounding environment, and its ionization state, but also on other factors, such as solvation, steric hindrance, hydrogen bonding, and formation of cyclic transition states [37,38,39]. Thus, although the largest portion of cysteines within cytoplasmic proteins is unreactive to H_2_O_2_, selected protein environments provide specificity for H_2_O_2_ signaling. The general chemical reaction with H_2_O_2_ is a nucleophilic attack, in which the deprotonated form of the cysteine side chain (-S^−^), a thiolate, attacks the peroxide bond (O-O) in H_2_O_2_ [40]. Stabilization of the negatively charged form of the cysteine is mediated by the presence of positively charged neighboring residues, frequently arginines, decreasing the local pKa [41,42]. The two-electron oxidation of a thiolate by H_2_O_2_ yields sulfenic acid, a naturally unstable modification [43] that can be the subject of several fates: (i) spontaneous reversal back to the thiolate, (ii) stabilization due to a favorable structural topology of the protein [44], (iii) enzymatic reduction by thioredoxins [45], or (iv) progression to further chemical oxoforms if the oxidant signal persists [46]. This onward oxidation can result in the formation of sulfinic (-SO_2_H) or irreversible sulfonic (-SO_3_H) acids. Besides, the formation of reversible disulfides, sulfenamides, S-glutathionylation, and other modifications can follow sulfenic acid formation, making secondary-derived thiol oxidation products from H_2_O_2_ further suitable to serve for signaling purposes [47]. The wide range of possibilities for modification of cysteines driven by the oxidation–reduction of thiolates is an important factor to diversify signaling, adding an incredible level of versatility to H_2_O_2_ as a second messenger. Furthermore, emerging evidence indicates that methionine, the second sulfur-containing amino acid, might provide an analogous redox-dependent system [48].

**Figure 2:**
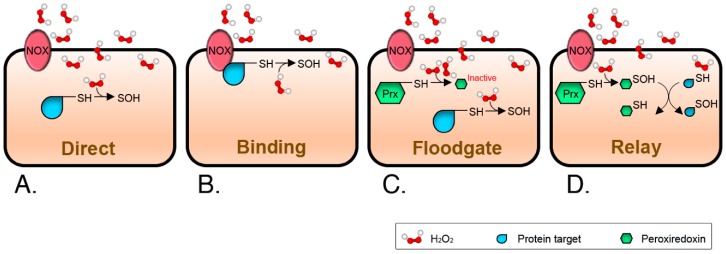
Main models for ROS signal transmission to specific cysteines. (**A**) The direct model presumes that redox targets (depicted as a blue teardrop) are constituted by proteins whose rate constant of oxidation is higher than that of the reaction of ROS with its major cellular scavengers, particularly peroxiredoxins. Direct chemical reaction of ROS with those targets is thus kinetically possible and leads to their oxidation. (**B**) The binding hypothesis proposes that H_2_O_2_ sources and targets are bound or in close proximity, allowing for site-localized oxidation to occur. Also in this case, ROS will directly oxidize targets but as a function of their relative proximity to the source instead of depending on their rate constant of reaction. (**C**) The floodgate model overcomes the apparent kinetic limitation of target oxidation by suggesting that overoxidation of peroxiredoxins (represented by green hexagons) permits further oxidation of other proteins with slower reaction rates. (**D**) Peroxiredoxins have been already shown to behave as relays, transmitting redox equivalents to targets during their oxidation–reduction cycle, and thus allowing for signal transduction. This model implies an indirect ROS effect on targets, as ROS will oxidize peroxiredoxins and will not chemically react with targets. In all cases, a plasma-membrane-bound NADPH oxidase (NOX, in dark pink) has been chosen as a representative source of ROS acting at the cellular surface. Note that the four possibilities are not mutually excluding.

Although the idea that H_2_O_2_-mediated signaling mostly relies on oxidation of specific cysteine switches is now firmly established, there are still unanswered questions about how the transmission of the signal proceeds. In vivo, the reactions of H_2_O_2_ with glutathione peroxidases (GPX) and peroxiredoxins (PRX) are most likely to occur due to the high rate constants of 6 × 10^7^ and 10^8^ M^−1^ s^−1^, respectively [49,50,51]. In comparison, the bulk of the currently identified redox-sensitive cysteinome presents very slow reaction rates, around 20 M^−1^ s^−1^ [42,51]. This means that H_2_O_2_ must either reach a high localized concentration, be produced for an extended time, or even both, to outcompete signal quenching. Thus, transmission of a redox signal from H_2_O_2_ to protein thiolates can theoretically occur mainly if: (i) the target cysteine has a rate constant equal to or higher than that of GPX or PRX (Figure 2A); (ii) the H_2_O_2_ source is close enough to the target protein to allow for site-localized oxidation (Figure 2B); (iii) the scavenging proteins are inactivated by over-oxidation, the so-called floodgate model (Figure 2C) [52]; or (iv) a highly reactive thiol protein acts as an intermediary, it is a signaling relay (Figure 2D) [33,53]. Apart from a few instances where PRX have been shown to be the relay transmitting the signal, evidence for these mechanisms is limited. This probably means that, as in those malicious questions in test exams, more than one answer can be true at the same time.

## 4. The Plasma Membrane as a Platform for Redox Signal Transmission

**Figure 3:**
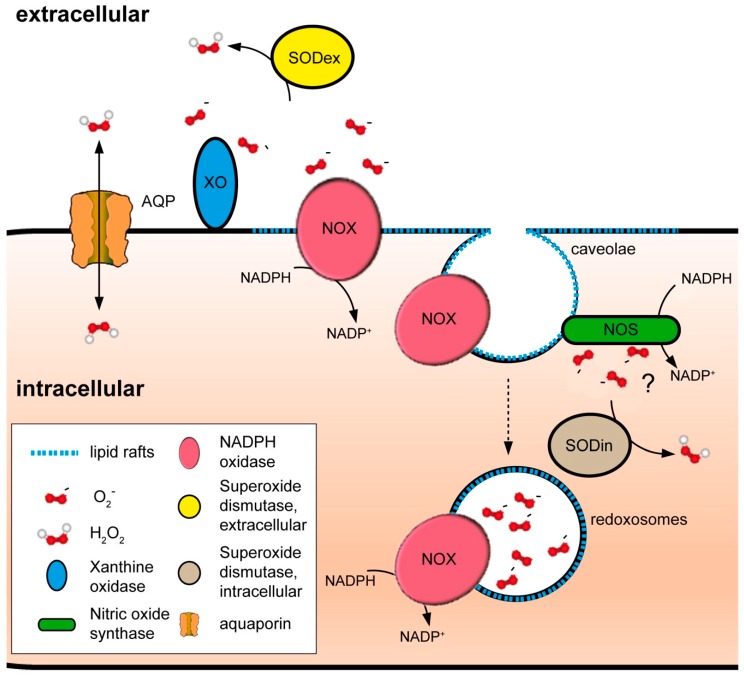
Cellular factors of plasma membrane redox signaling. As integral membrane proteins, NADPH oxidases (NOX, dark pink) are present either on lipid rafts (dashed blue line) or in caveolae, releasing their product superoxide (O_2_^•−^) into the extracellular space. Upon activation of NOXes, these enzymes can be also internalized into forming redoxosomes where they are still functional. Besides, extracellular O_2_^•−^ can be generated by the activity of an extracellularly linked xanthine oxidase (XO, blue). In its uncoupled state, NOS enzymes also produce O_2_^•−^ in the vicinity of caveolae, but since there is a certain controversy on whether this happens in non-pathological conditions, the fact is indicated with a question mark. Nevertheless, extracellular (SODex, yellow) or intracellular (SODin, brown) superoxide dismutases convert O_2_^•−^ into hydrogen peroxide (H_2_O_2_) depending on the localization of the sources. H_2_O_2_ is transported inside or outside the cell by specialized aquaporins (AQP, orange channel), following cellular needs.

Redox chemistry cannot be conceived separately to the notion of redox compartmentalization. Current evidence well-supports the existence of distinct redox environments within the cell, such as in mitochondria and endoplasmic reticulum (ER) with respect to the cytoplasm [33,54]. However, while much attention has been directed to the ROS-producing enzymes installed in those organelles, substantially less has been dedicated to the plasma membrane, as an entity preserving the low background of ROS that allows for specific signaling events and as a dock whose architecture facilitates the concentration of many ROS-related proteins (Figure 3). Indeed, not only integral membrane proteins, such NADPH oxidases (NOX), exert their activity at the plasma membrane [55]. Also, other redox enzymatic systems, such as xanthine oxidase (XOR) [56] and nitric oxide synthases (NOS) [57], generate ROS at the outer or inner part of the membrane, respectively. Moreover, independently of the leaflet where ROS are produced, bidirectional H_2_O_2_-transporting proteins assure the correct distribution and delivery of the signal [54,58], and further control of redox processes is guaranteed by the localization of scavengers in the vicinity of the plasma membrane [59,60,61,62]. Furthermore, the plasma membrane is the starting point of processes enabling redox signaling inside the cell. For example, specific endosomes, termed redoxosomes, are formed from invaginations on the plasma membrane, allowing for highly spatially localized intracellular ROS production [63].

Therefore, from the perspective of redox biology, the plasma membrane can be defined not merely as an instrumental physical barrier protecting cells from oxidative insults, but also as an organizing center that both directs and maintains redox signal specificity. To explain the manifold implications that this concept has, we give examples for several relevant processes outgoing from a general description of the membrane’s structure.

### 4.1. The Plasma Membrane: More Than Lipids

Referring to the classical membrane fluid mosaic model of Singer and Nicholson, biological membranes are bilayers of phospholipids that are organized in a hydrophobic center and hydrophilic outer leaflets and therefore serve as diffusion barriers. To allow for a selective interchange of molecules or information, amphipathic proteins are solved in the lipid matrix [64]. Since the late 1990s, this simple view of lipids as solvents for proteins has been overcome and a more complex picture has been accepted. Thus, as result of an asymmetrical distribution of specific lipids, membranes are further organized in lipid rafts (LRs), defined by being detergent-insoluble sphingolipid- and cholesterol-rich domains [65]. These regions have been demonstrated to be active structural signaling organizers rather than simply building blocks, being either enriched with specific components or enabled with the capacity to recruit them upon stimulation. Many transmembrane proteins have been shown to have affinity for LRs, including receptors, ion channels, and transporters, while cytoplasmic proteins associate to LRs typically by post-translational modifications, such as glycosylphosphatidylinositol (GPI)-anchoring, palmitoylation, and myristoylation [66,67]. Indeed, their capacity either to bring together different proteins that cooperate to transfer a signal, or to physically sequestrate others to block unspecific signaling, is crucial to allow for signaling processes [68]. Importantly, a large group of redox-related proteins have been found in LRs, foremost NOXes, whose downstream signaling is interrupted by drugs that disrupt LRs, and phosphatases, the earliest identified redox targets [69,70]. Moreover, some evidence suggests that the formation of LR domains may be itself altered by ROS, either directly by enhancing the activity of enzymes that promote LR clustering [71] or indirectly through their effects on the synthesis of lipids, such as ceramide or cholesterol [72,73].

A special type of LR are caveolar rafts, membrane invaginations generated by caveolin proteins [74]. At least three caveolin isoforms have been identified: caveolin-1 and caveolin-2 are expressed in most cell types, while caveolin-3 is specific of muscle cells [75]. Caveolins not only structurally define caveolae, but act as protein scaffolds to facilitate protein interactions in a restricted area of the plasma membrane. Notably, caveolin-1 has been shown to be phosphorylated by redox-sensitive kinases, such as Fyn, Abl, and Src, in response to ROS [76,77,78], and this modification is able to change its binding partner profile [79,80]. Furthermore, increasing evidence relates intracellular ROS levels to caveolin-1 expression [81], repression of its degradation [82], and membrane trafficking [83], suggesting feedback regulatory processes. Remarkably, caveolae structures have been also recently linked to the formation of redox-active endosomes, so-called redoxosomes. These single-membraned organelles generate ROS in an enclosed environment, thus facilitating co-localization of ROS generators and targets and preventing non-specific ROS-dependent damage reactions [63,84,85]. In mammalian systems, several stimuli have been identified to result in the formation of such redoxosomes, among them interleukin-1-β (IL-1β), tumor necrosis factor α (TNFα), and hypoxia=reoxygenation (H=R) [86,87]. In all those processes, members of the NOX family were identified as the source of O_2_^•−^ generation within the redoxosome, suggesting a mechanistic conservation of signaling [85]. Intriguingly, localization of some receptors either to the plasma membrane or to endosomes modulates their potential to be activated, thereby regulating which downstream cascades are turned on. As an example, EGF receptor (EGFR)-triggered pathways can be either modulated depending on the presence or absence of endocytosis of the activated EGFR, or independently of localization and activation at the plasma membrane, since the active signaling of EGFR is taking place in the redoxosomes [88,89,90,91]. The discussed underlying mechanisms are divergent ligand-binding capacities due to different lipid compositions in endosomes or fusion of redoxosomes with vesicles harboring second effectors [92]. Besides the described caveolin-dependent formation of redoxosomes, there are indications for a probable clathrin-dependent process. In a recent study dealing with *Clostridium difficile* toxin B (TcdB)-induced necrosis in diarrhea, the authors speculate about internalization of the toxin together with p22^phox^, a critical component of some NOXes, to clathrin-coated vesicles, resulting in the formation of redoxosomes, ROS overproduction, and tissue damage [93]. In parallel, the internalization of NOX homologs has been shown to be clathrin-dependent in plants [94].

Apart from LR and caveolae, polyphosphoinositides (PPIn) form anchor points specifically associating proteins to the cytoplasmic leaflet of eukaryotic membranes, and therefore providing platforms for cellular signaling. Various isoforms of PPIn exist, resulting from differential phosphorylation of the inositol ring of phosphatidylinositol (PtdIns) [95]. These PPIn can be recognized by several highly conserved lipid-binding domains in proteins, such as the PH, FERM, FYVE, and PX domains, and thus regulate protein localization impacting its activity [96,97]. Regarding redox signaling, PX–PPIn interactions are crucial to allow the activation of several NOX isoforms [98,99,100,101]. Further, several studies have reported an impact of H_2_O_2_ on PPIn formation and hydrolysis [102,103,104,105], probably as result of its known effects on kinases and phosphatases, such as PTEN [106].

Despite that all of the above-described constituents of the plasma membrane have been shown to house important ROS-related systems, their way of facilitating redox signaling events might be quite diverse due to their different dynamics: while both non-caveolar LR and PPIn-anchors are continuously facing changes due to clustering or declustering of components or phosphorylation and dephosphorylation events, the composition of caveolar LRs is stable and hardly rearranged and might only change due to endocytosis events or fusion with vesicles. Thus, it is not surprising that differential targeting of ROS-producing enzymes and redox targets to these lipid-interaction platforms mediates distinct signaling pathways to orchestrate different cell responses.

### 4.2. NADPH Oxidases and Peroxiporins as a Generator–Facilitator System on the Plasma Membrane

The seven members of the human NADPH oxidase (NOX) family are widely recognized as the most important sources of signaling-competent H_2_O_2_. All of them have been identified at the plasma membrane of different cellular types in several tissues (Table 1), allowing for both general and cell-type-specific redox-dependent pathways to occur [13].

Broadly speaking, NOXes catalyze the oxidation of NADPH and the reduction of molecular oxygen via a highly conserved flavocytochrome core: six transmembrane domains hold a heme cluster that transfers electrons from NADPH through to a membrane [107]. A second membrane-spanning subunit, p22^phox^, provides stability to the complex in the majority of the isoforms (NOX1 to 4) [108]. Due to structural differences, the NOX family is further divided in ‘true’ NOX enzymes and dual oxidases (DUOX). In the DUOX case, an additional seventh transmembrane domain is linked to an N-terminal peroxidase-like domain via a short cytosolic bridge to allow for direct generation of H_2_O_2_. In contrast, the final product of NOX1, NOX2, NOX3, and NOX5 is O_2_^•−^. To ensure H_2_O_2_ production by these NOX family members anyhow, they cooperate in a finely balanced way with SOD enzymes [109,110]. As an exception to the general theme, NOX4 can be cited. This enzyme is—in contrast to all other family members—constitutively active without the need for stimulation [111]. Furthermore, it directly generates H_2_O_2_ despite lacking the DUOX-typical domain and it has been described to be mainly an ER-resident enzyme [112]. However, some controversy exists and several studies also report NOX4 localization to many other sites in the cell, including the nucleus and the plasma membrane [113,114].

The other ‘true’ NOXes are plasma membrane proteins that localize both to caveolar and non-caveolar rafts [74]. Some NOX enzymes require a subset of regulatory proteins for full activation of their catalytic subunit [132]. The recruitment of these regulators can be induced via diverse stimuli, allowing for a precisely timed release of H_2_O_2_ and resulting in NOX-isoform-specific outcomes [133]. Thus, the NOX1, NOX2, and NOX3 complexes are considered to be inactive or to display low levels of ROS production in membrane LR until their cytoplasmic activators are recruited [134,135]. In the case of NOX2, the best-studied member of the family, ligand-induced stimulation causes translocation to the plasma membrane of the pre-associated regulatory subunits p40^phox^, p47^phox^, and p67^phox^, where they are stabilized by PX domain–PPIn interactions [99]. Another necessary activator, the small GTPase Rac, directly interacts with the membrane through a geranyl lipid modification [136]. As cholesterol depletion impacts more on membrane subunit retention and complex activation rather than on activator translocation [137,138], it has been proposed that localization of all the NOX2 constituents to LRs is a sequential step to membrane binding of the regulatory proteins directed to solve the intricacies of bringing together all of the components in the correct spatial orientation to achieve proper assembly and full activation [134]. Similarly, activation by the NOX1 and NOX3 regulator, NOXO1, is also preceded by a PX–PPIn interaction of the subunit [98]. Instead, for NOX5, which depends on a EF-hand Ca^2+^-sensitive region for activation rather than on interaction with regulatory proteins, it has been shown recently that its N-terminal region binds to phosphatidylinositol 4,5- bisphosphate (PtdIns(4,5)P2), promoting localization to cholesterol- and caveolin-rich subregions at the plasma membrane [100,101]. Furthermore, it has been speculated that PtdIns(4,5)P2 has an additional function in co-localization of NOX5 with its target F-actin [139]. Finally, it has been shown that DUOX proteins require the presence of two maturation factors, DUOX activator 1 and 2 (DUOXA1 and DUOXA2), to be properly processed and exit the ER to the plasma membrane [140,141]. Once there, DUOXes are activated by Ca^2+^ mobilization and binding to EF-hand motifs, but whether they are targeted to any kind of LR is currently not known. However, phospholipase C β (PLCβ) and protein kinase Cα (PKCα), two factors inducing Ca^2+^-mediated activation of DUOXes, are enriched in lipid rafts [142,143], a fact that would predict DUOXes localization to the same site. In support of this hypothesis, a recent publication has linked LR-dependent Ca^2+^ signaling to DUOX-mediated ROS generation in *Drosophila* during pathogenic infection [144].

Importantly, the presence of NOX proteins at a particular region of the plasma membrane is not enough to explain the efficiency of their ROS-derived signaling. Thus, a finding of great significance to understand transmission of redox messages may lie in the evidence that H_2_O_2_ can be transported in a regulated manner across biological barriers, and that variations in permeability modulate its downstream intracellular effects [26]. For instance, although the redox-driving activity of plasma-membrane-bound NOX downstream of receptor activation is beyond doubt [145,146,147], known cytosolic targets of the NOX-generated H_2_O_2_ do not display reaction rates defeating that of PRX [148,149,150]. Furthermore, non-directional diffusion of H_2_O_2_ would compromise the possibility of any type of site-localized oxidation, including the floodgate model. PRX-based relays and co-localization with target proteins in intracellular structures, such as redoxosomes, are still possible in this context, but insufficient to explain the whole bunch of NOX-regulated processes.

Peroxiporins are now recognized as a further subclass of the aquaporin (AQP) protein family of membrane channels that are able to permeate H_2_O_2_ in addition to water or glycerol [151], whose functional characterization has opened new interesting perspectives on the possible mechanisms of NOX-mediated redox signaling. The net flow of solute through AQPs is dictated only by concentration gradients, implying that they are not active pumps or exchangers like Ca^2+^ transporters, but bi-directional passage facilitators. Movement of substrates through bilayers has been calculated for water to be accelerated by 5–50-fold in comparison to passive diffusion [152]. Assuming that the size and electrochemical properties of H_2_O_2_, including its capacity to form hydrogen bonds (fundamental for traversing the AQP pore), are quite similar to water [153], it is expected that H_2_O_2_ transport velocity ranges amongst similar parameters. The peroxiporin functional category currently comprises AQP3 [154], AQP8 [58,155], and AQP9 [154,156], whereas AQP11, an ER-resident AQP, has been also suggested to have a peroxiporin role [157]. In this last case, the idea of the existence of two H_2_O_2_-transporting channels, one situated in the plasma membrane and one in the ER, is somewhat appealing, as it resembles very much the scheme followed by Ca^2+^, in which the second messenger is stored in membrane-protected compartments in order to exclude the molecule from the cytoplasm, allowing for spatiotemporal specificity on signaling in restricted areas of the cell.

Following these lines, and considering the potential harmful consequences of uncontrolled release of H_2_O_2_ into the cytosol, it was expected that some type of redox-dependent regulation was operating on peroxiporins. Indeed, early studies indicated that AQP-mediated water transport could be inhibited by oxidative gating [158,159,160]. Two alternative mechanisms were proposed: (i) H_2_O_2_ directly oxidizes and thereby inhibits aquaporins; or (ii) H_2_O_2_ acts as a signaling molecule activating a pathway that ultimately leads to channel closure. However, no inhibitory effect of H_2_O_2_ on the conducting activity of individual AQPs heterologously expressed in yeast or *Xenopus* oocytes could be recorded [58,161,162,163]. Remarkably, when investigating membrane permeability to H_2_O_2_ in conditions of cell stress, Medraño-Fernandez et al. uncovered that a reversible redox-based mechanism modulates the transporting capacity of the peroxiporin AQP8 [26]. Gating involved the combined and coordinated action of H_2_O_2_ and the gasotransmitter H_2_S in a two-step process: First, a reactive cysteine inside the extracellular vestibule of the pore (C53) is primed by sulfenylation through the passage of the transported H_2_O_2_ molecules. Then, the moiety is modified by persulfidation induced by the activation of the H_2_S-producing redox-sensitive enzyme cystathionine-β-synthase (CBS), which is situated in the vicinity of the cytosolic mouth of the channel [164]. The latter modification elongates the side chain of C53 in a way that a histidine (H72), situated in the narrowest part of the pore, is displaced from its original position, leading to channel blockade. The system has been hypothesized to function as a sensor of the levels of H_2_O_2_ molecules crossing the plasma membrane [164]. Interestingly, C53 of AQP8 is highly conserved in all known peroxiporins [26,164], suggesting that a similar mechanism of gating could be operating in all of them.

**Figure 4:**
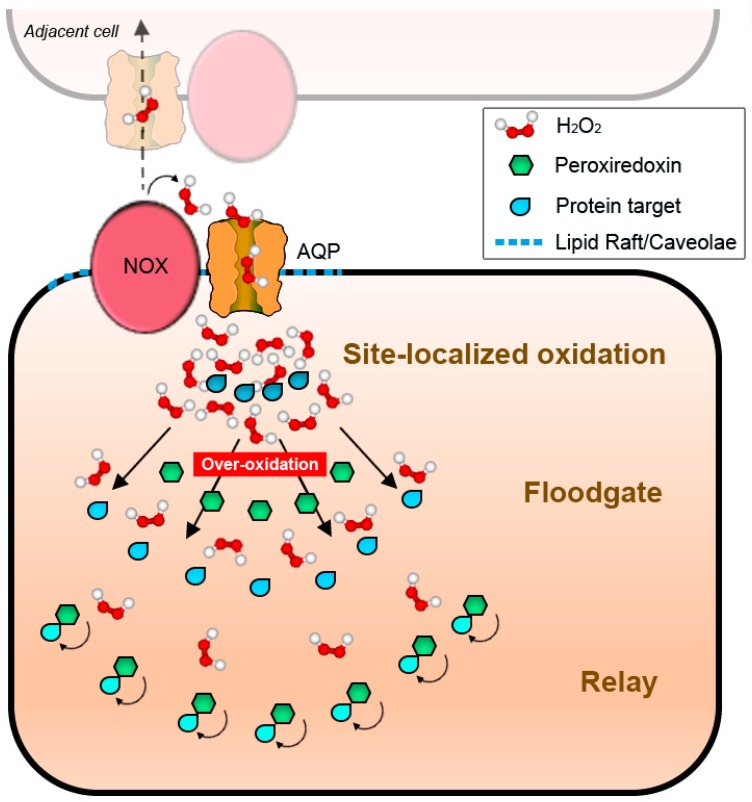
Putative mechanisms adopted by cells to achieve redox signal transmission after peroxiporin-mediated routing of ROS through the plasma membrane. The NOX/AQP system, situated in privileged lipid platforms for signaling (blue dashed line), offers a new perspective to re-interpret the models for redox signal transmission to intracellular targets (depicted as blue teardrops): site-localized oxidation of vicinal proteins will be favored by strategical positioning near the cytosolic mouth of the channel, possibly by localizing them to lipid raft domains, while further proteins would be reached either directly by overoxidation of peroxiredoxins (represented as green hexagons) or indirectly via peroxiredoxin-mediated relays. The particular disposition of NOXes releasing ROS to the extracellular space will also induce similar pathways in a peroxiporin-equipped adjacent cell (upper part of the scheme), thus allowing for a coordinated response. The figure has been organized as a cascade for clarity. However, all modes of signal transduction may be not hierarchical and concur simultaneously.

The fact that peroxiporins facilitate H_2_O_2_ transport across the plasma membranes gives a new perspective to redefine the hypotheses on ROS-derived signal transduction previously enunciated (Figure 2). Regulated H_2_O_2_ transport through channels provides directionality to signals, potentiating the formation of cytosolic gradients with the capacity to restrain redox reactions as a function of site-localized concentration. In this scenario, all four possibilities of redox signal transmission would be feasible, either as independent, contemporary, or hierarchical events. Together, these may result in the activation of different sets of signaling layers to allow for an appropriate response to the stimulus arriving from the plasma membrane (Figure 4): discrete fluxes of H_2_O_2_ could oxidize strategically positioned proteins in the immediate proximity of the cytosolic mouth of the transporter (as CBS) [164], even if their velocity of reaction is slower than that of PRX. Higher rates of H_2_O_2_ transport would, on the other side, over-oxidize vicinal proteins, including PRX, permitting floodgate model-like signal transmission. Finally, relays would be possible depending on the localization of the putative intermediaries: low H_2_O_2_ concentrations would allow for the modification of proteins near the channel via local PRX, while more distant PRX would be oxidized and act as relays whenever fluxes exceed proximal scavengers and diffuse to further positions within the cell.

Notably, current research further points to a functional interaction of NOX enzymes with H_2_O_2_-transporting channels: in mouse primary keratinocytes, co-immunoprecipitation of NOX2 with AQP3 has been shown, demonstrating that these two proteins act in concert as H_2_O_2_ producer and facilitator to allow for successful redox signaling [165]; analogously, a similar NOX2–AQP8 cooperation axis has been described on B-cells [166]. Moreover, it is important to underline that all putative components of the above-described redox circuitry, H_2_O_2_-stimulating receptors [167], NOXes [13], extracellular SODs [168], peroxiporins [169], peroxiredoxins [59], and cysteine targets [170,171,172,173], have been identified in LRs. Even accumulation of homocysteine, the principal CBS substrate, in mice deficient for this enzyme has been linked with oxidative-stress-mediated tissue injury due to exacerbated ROS production by NOXes in LRs [174]. This last observation was attributed in the past to the promotion of LR stability by homocysteine; however, in light of the uncovered functions on controlling H_2_O_2_ permeability of CBS, it is also conceivable that the absence of the enzyme negatively impacts termination of ROS-driven signaling in LRs, and that both mechanisms cooperate to cause damage.

Altogether, these data would imply that special domains in plasma membranes are truly operating as a regulatory hub for NOX-derived signaling, linking external stimulus to redox responses, and serving as a platform to orchestrate differential activation of H_2_O_2_-mediated pathways. Moreover, the regional association of the ROS-related machinery to particular areas of the plasma membrane may favor cell-to-cell signaling or functional coordination with similar signaling platforms situated in vicinal cells, with or without direct protein–protein interaction (Figure 4, see upper part).

### 4.3. Other Sources of ROS at the Plasma Membrane

As anticipated before, not only NOXes exert their activity at the plasma membrane. For space constraints, we will dedicate this section only to two further predominant systems in terms of ROS production, xanthine oxidase and nitric oxide synthase, though other minor ROS producers, such as certain lipooxygenases [175] and cyclooxygenases [176], have been reported to be localized in the vicinity of the plasma membrane, at least in part of their life cycle.

*Xanthine oxidase*. In contrast to aquaporins and NOXes, which are present in several isoforms, the xanthine oxidoreductase (XOR) is a single enzyme mainly expressed in liver, intestine, kidney, and lactating mammary gland epithelial cells, but generally present in low levels in all human tissues [141,142,143,144,145]. Furthermore, its activity is organ-specific. It consists of two identical subunits forming a 300-kDa homodimer containing two iron–sulfur redox centers, a molybdopterin cofactor (Moco), and a flavin adenine dinucleotide (FAD) cofactor [146]. The Moco site is responsible for the main catalytic process of the enzyme, the oxidation of hypoxanthine to xanthine and xanthine to uric acid, but can also metabolite various endogenous substances and xenobiotics [147]. XOR is localized on two main sites in a cell: either in intracellular vesicles, which might be storage points of XOR [148], or the surface of plasma membranes. In the latter case, most of XOR is particularly localized in membrane regions facing closely neighboring cells, suggesting a role in inter-cellular signaling [148,149,150]. Nevertheless, no localization to lipid rafts or caveolae has been shown so far [79]. Interestingly, the mammalian XOR undergoes various changes, thereby switching from a NAD^+^-dependent xanthine dehydrogenase form (XDH) to the oxidase form (XO) [151]. This formation process is mediated by reversible thiol oxidation (XDH) or limited proteolysis (XO) and results in the generation of two completely different enzymes: whereas the FAD site of XDH converts NAD^+^ to NADH, XO catalyzes the generation of O_2_^•−^ and H_2_O_2_ from molecular oxygen [152,153]. Furthermore, hypoxic conditions trigger even more drastic changes: the affinity for nitrites increases under these conditions, resulting in the generation of NO and reactive nitrogen species (RNS) [148]. Notwithstanding, the relations between XOR forms and ROS generation are not absolute: XDH might be less efficient than XO in reducing molecular oxygen, but in the absence of its preferred substrate NAD^+^, the Km value for oxygen for XDH is 600% higher than for XO [154]. Therefore, even a general increase of XOR activity might give rise to higher ROS production [148]. Due to these different XOR variations and the resulting enzymatic products, this enzyme is described to have both cytotoxic and pro-inflammatory activities alike as to trigger pro- and antitumorigenic effects [152]. In general, it is suggested that the main ROS generated by XOR is the O_2_^•−^, and thus its activity has mainly been linked to tissue damage and bacterial killing within the immune response. A typical example is the release of XOR from capillary endothelial cells into the blood in response to diseases, such as meningitis and malaria, followed by the establishment of a microvascular inflammatory response [148,155,156,157]. Intriguingly, under inflammatory conditions, O_2_ and pH values are significantly decreased, resulting in the almost exclusive production of H_2_O_2_ by XOR, which give rise to possible roles of XOR signaling regulation during inflammation [158]. Indeed, a crosstalk between inflammatory pathways and XOR signaling has been shown: cytokines, such as tumor necrosis factor α (TNFα), interleukin-1 (IL-1), and interferon-γ (IFN-γ), regulate XOR activity by expression regulation, since the XOR 5’ region exhibits binding sites for all three cytokines [159,160]. At the same time, there is evidence that INF-γ modifies XOR at the post-translational level, increasing thereby its activity up to 8-fold [160,161]. The other way around, ROS released by XOR increases the expression of NF-κB, implying an intensive crosstalk of both proteins and the corresponding pathways [162,163]. Nevertheless, the impact of XOR in signaling shown so far is mainly based on its product NO and on the generated ROS species O_2_^•−^ and H_2_O_2_. NO was, for example, shown to regulate the ryanodine receptor and the Ca^2+^ ATPase in the heart, two factors of Ca^2+^ signaling [164,165]. Since these changes occur on reactive thiols in the corresponding proteins, it would be interesting to elucidate possible changes in signaling outcome dependent on the current form of XOR and the corresponding products in detail [166].

*Nitric oxide synthase (NOS)*. NOSes are a family of enzymes responsible for the production of NO and L-citrulline from L-arginine and O_2_ through the binding of the critical co-factor tetrahydrobiopterin (BH4). Even if NOS-mediated production of nitrogen species is undoubtedly of great importance, space constraints limit us to comment only on the role of these enzymes as generators of ROS, and we refer the interested reader to other excellent reviews on the matter [177,178,179]. In this sense, aside from NO, NOSes also catalyze an “uncoupled” reaction that leads to O_2_^•−^ production [180,181,182]. The word “uncoupling” indeed refers to the principal situations in which this phenomenon occurs, that is, when NOSes are not coupled either with its cofactor or with its substrate. The ultimate causes of NOS uncoupling are not completely understood, but O_2_^•−^-induced oxidation of BH4, changes in NOS phosphorylation, S-glutathionylation of the enzyme, and disruption of its functional structure have been suggested to play a role in the activity [183]. Three isoforms of NOS have been identified: neuronal NOS (nNOS, type I), inducible NOS (iNOS, type II), and endothelial NOS (eNOS, type III) [182,184]. Activity of NOSes is dynamically modulated by differential targeting of the enzyme to diverse subcellular localizations. For example, in resting endothelial cells, most eNOS protein is localized to caveolae by lipid modifications [185]. Similar to NOX subunits, myristoylation at the eNOS N-terminus appears to be an early event resulting in translocation to the plasma membrane, whereas palmitoylation of the enzyme leads to caveolar targeting [186,187]. This process can be reversed by agonist stimulation, causing eNOS intracellular trafficking by depalmitoylation [188], a process that has been suggested as a negative feedback mechanism for downregulating eNOS activity after signaling [179]. Importantly, NOSes are also regulated by caveolin binding (caveolin-1 for eNOS and iNOS and caveolin-3 for nNOS in skeletal muscle), but this interaction is not required to locate NOS to caveolae [177,189,190]. Instead, caveolin inhibits eNOS and nNOS activity by blocking the access of L-arginine to these proteins. Further, caveolin acts as an allosteric competitor for calmodulin, whose Ca^2+^-dependent binding is a crucial event for activation of these enzymes. For iNOS, the inhibition mechanism is quite diverse: in this case, caveolin promotes iNOS degradation by a proteasome-dependent pathway to terminate signaling [191]. Although it is clear that this NOS inhibitory/stimulatory mechanism and consequent NO production are facilitated in caveolae, it is unknown so far whether the same cycle and the same interactions affect O_2_^•−^ generation. However, there is evidence for a controlled uncoupled process for eNOS and nNOS, governed as well by protein–protein interactions in the context of caveolae. This would suggest that, although classically related to pathologic stages, uncoupling may well also have a signaling role in physiological conditions [192]. Since both NOXes and NOSes are localized to caveolae, it will be of paramount interest to investigate how these two facts crosstalk and which impact on signaling derives from them. Interestingly, solely the chemical interplay between NO and O_2_^•−^ will be not necessarily be the only answer: analysis in vitro suggests that S-nitrosothiol formation can be promoted by conjoint NO/ O_2_^•−^ reactions on targets when both species are generated simultaneously and at low levels [193,194]. As caveolae are preferential sites for S-nitrosylation [195], it can further be speculated that the shut-down of uncoupled NOS activity is a necessary step for NO-driven signaling stimulated by NOXes, aimed to avoid the destructive effects of excessive O_2_^•−^ production in the microdomain.

## 5. Dysregulation of Redox Signal Transmission through the Plasma Membrane: Impact on Disease

When ROS are produced in large amounts and bypass the antioxidant capacity of the cell, maladaptive oxidative stress is triggered [196]. As signaling molecules, it is not counterintuitive that dysfunction in ROS production can be a major source of malfunctioning during transduction of information both towards the inside and the outside of the cell. The particular disposition of NOXes at the plasma membrane, facing the extracellular space, and the bi-directional nature of peroxiporins, only reinforce this notion. Therefore, oxidative stress must not be seen as an isolated event, damaging macromolecules in a single cell or causing inadequate activation of intracellular pathways. Most likely, all nearby structures will be damaged, and spurious activation of redox-dependent cellular pathways should be also expected in neighboring cells. A clear example is provided by uncontrolled inflammation, which is not even a cell-type-restrained effect, but a systemic problem disturbing the normal functioning of several cell types residing in the same area.

Indeed, diseases that course with an inflammatory component provide the best model to appreciate to what extent the correct structuration of plasma membrane and incorporated redox signaling systems, from lipids to enzymes, is fundamental to assure timed and reliable signal transduction and to restrain oxidative damage. To illustrate this aspect, we have selected examples of redox imbalance in various tissues involving the NOX/AQP system, as the major source of potentially detrimental ROS in the area.

### 5.1. Immune System Diseases, NADPH Oxidases, and Peroxiporins

The enormous amount of data about how ROS influence the immune system makes it pretty difficult to offer an integrative and non-confusing view on ROS involvement in normal and pathological immune responses. Broadly speaking, it seems that heterogeneity can be solved by assuming a dual role of ROS in immune-related disease: beneficial at initial stages, but perpetuating the progression of the disease by fostering chronic inflammation [197]. Additionally, as discussed before, when it comes to ROS signaling, an adequate balance of their levels is the key to sustain homeostasis.

One of the most trademark characteristics associated with phagocytes is the activation of a powerful oxidative burst during their antimicrobial functions. This striking feature captured the attention of numerous researchers, leading to the identification of the first ROS-generating complex, NOX2 [198]. Since then, it has not only been confirmed that NOX2 produces ROS on purpose, but also that localization and timing of ROS production seems to be highly organized to respond to different challenges: while upon particle phagocytosis short limited NOX activity occurs at the phagosomal membrane, priming leads to prolonged NOX2 activity and ROS release mostly on the plasma membrane, directing ROS into the extracellular space [199,200,201]. Thus, killing of foreign invaders can run in parallel with other ROS-coordinated pathways, such as inflammation, modulating the strength of the response to be consistent with the scale of the stimulus.

The strongest evidence of the broad importance of NOX2 was gained in studies analyzing its loss of function. Chronic granulomatous disease (CGD) is a rare inherited immunodeficiency caused by mutations in one of the genes encoding for NOX2 complex components [202,203,204,205]. In this syndrome, a defective activation of NOX2 leads to a strongly diminished ROS production even if pathogens can be efficiently internalized [206,207], possibly resulting in life-threatening bacterial and fungal infections in CGD patients [208,209]. Though the link between absence of ROS production and defective killing mechanisms is rather obvious, the fact that hyperinflammation and oxidative damage has been frequently observed in CGD patients has not been so easy to conceal. The prevailing hypothesis is that non-degraded phagocytosed material accumulates in phagosomes, thereby increasing the pro-inflammatory phenotype. Nevertheless, this does not explain why CGD macrophages are showing reduced efferocytosis [210]. Furthermore, CGD macrophages are defective on producing anti-inflammatory mediators, preparing a fertile ground to tissue damage [211]. The discovery that ROS can be efficient second messengers on signaling pathways shed light on these, on the first glance, counterintuitive findings, and explained how alterations in ROS levels are able to favor inflammatory disorders [212,213,214]. Moreover, expression of certain innate immune receptors, such as TLR5, is reduced on the surface of CGD neutrophils [215], indicating that redox-sensitive signaling elements control either the transcription of specific immune-related genes or their trafficking to the plasma membrane. In support of this view, myeloperoxidase (MPO) deficiency is rarely associated with serious infections [216], even if MPO products are faster and more potent as antimicrobial agents than ROS [201], suggesting that the impaired immune response is not only the result of the lack of a defensive mechanism but a consequence of ROS-derived signaling.

In the last decade, this vision has gone beyond innate immunity, extending NOX2-generated ROS functions to adaptive immune-related processes [217,218,219]. For example, NOX2 activity has been related to functional communication between the innate and adaptive arms of the immune system [220], making CGD carriers prone to develop autoimmune diseases, such as polyarthritis, lupus erythematosus, and Crohn-like inflammatory bowel disease [218,221]. Several NOX2-dependent processes have been described to provide successful antigen presentation by dendritic cells: NOX2-dependent O_2_^•−^ generation allows for antigen presentation of MHC class I to CD8^−^ T-cells [222,223,224,225,226,227] and further inhibits the degradation of antigen in the phagosome [227,228]. A striking similar secretory deficiency as in CGD has been described in mice B-lymphoma cells with a downregulated expression of the peroxiporin AQP8 [166]: unlike their normal counterparts, AQP8-silenced cells continue to express IgM on their surface after LPS stimulation and secrete less IgM polymers. From these findings, it is deduced that AQP8 might channel NOX2-generated ROS to promote B-cell differentiation into secreting plasma cells [166]. This goes in line with the fact that CGD patients have a reduced memory B-cell count, which is reflected in a decreased capability to maintain a long-term memory defense against pathogens [229,230].

Even if little is known about the expression of the different peroxiporin isoforms in immune cells, some evidence indicates that all peroxiporins may have representation in one or another cell type. Thus, human macrophages express AQP3 [231] and possibly AQP9 [232]; B-cells upregulate AQP3 and AQP8 during B-cell differentiation [166,233]; T-cells and immature dendritic cells bear at least AQP3 [233,234], while only AQP9 seems to be present in neutrophils [235]. Most available reports in the literature have constantly implicated AQP3 and AQP9 with migration processes of different immune cells. AQP3, for example, is essential for chemokine-dependent T-cell migration and to establish a sufficient immune response [234], whereas AQP9 knock-out mice show reduced neutrophil migration to the bacterial product fMLP [236].

Altogether, these data are consistent with the concept of a plasma membrane redox signalosome as a platform for signaling, as ROS producers, transporters, effectors, and inhibitors are jointly gathered in a restrained space, facilitating a coordinated and timed response.

### 5.2. Inflammatory Bowel Disease, NADPH Oxidases, and Peroxiporins

Inflammatory bowel disease (IBD) is the common term for a group of recurring inflammatory conditions in the gastrointestinal tract [237,238]. The main IBD disorders are Crohn’s disease (CD), characterized by inflammatory patches and deep ulcers all along the digestive tract, and ulcerative colitis (UC), which is limited to the mucosal lining of the colon and appears in a continuous pattern. The symptoms can be very similar, typically including abdominal pain, severe diarrhea, fatigue, weight loss, and malnutrition [239]. Although the mechanisms underlying IBD still remain elusive, it is widely accepted that an over-reaction of the immune response in a genetically susceptible background leads to oxidative stress and damage of the intestinal epithelium. The course of the disease further produces maladaptation to environmental factors and intolerance to the residing microbiota, while facilitating the entry of external invaders [240,241], inducing an auto-amplifier state that re-activates the immune system and creates a perfect storm [242]. Thus, the primordial cause of IBD seems to be a malfunction of host defense responses in an organ particularly well-armed to face external threats.

An imbalance in redox homeostasis starting at the plasma membrane may also be pivotal in IBD pathogenesis, as it has been reported that a critical window of optimal ROS production is necessary to permit the constant renewal dynamics of the intestinal lining [243], and that increases in ROS levels cause upregulation of genes involved in innate and adaptive immune responses specifically in the gastrointestinal tract [244,245]. Thus, it is not surprising that dysfunction of NOX enzymes has been now investigated as a potential risk factor for IBD. Both NOX1 and DUOX2 are expressed in the gut epithelium, but with differential distribution. Whereas NOX1 is expressed at high levels only in the colon [246], the DUOX2 protein is located at the apical membrane of enterocytes, with maximal expression in the tip epithelium of ileum, cecum, and colon [247]. This asymmetrical confinement of NOX isoforms seems to reflect their differential functions: while NOX1 fulfills eminently a regulatory role on both Wnt/ β-catenin and Notch pathways controlling proliferation of stem and progenitor epithelial cells [248,249], DUOX2 coordinates the innate defense in the gut of mammals by controlling the circuitry driven by interleukin receptor activation [250,251].

On the peroxiporin side, only the presence of AQP8 and AQP3 has been profusely documented along the gastrointestinal tract, while AQP9 expression has been restrained to a small population of mucus-secreting globet cells in the ilium and duodenum [252]. AQP8 transcript is expressed both in the small and the large intestine, principally in the duodenum, jejunum, and colon [252,253]. Its subcellular distribution at subapical regions of epithelial cells has been mostly related to its putative transephitelial water-transporting capacity. Notably, when analyzing the AQP8 knock-out mice, it was determined that deficiency in this AQP had little effect on the colonic fluid absorption, fecal dehydration, or in the small intestine agonist-stimulated fluid secretion [254]. Instead, in a drug-induced colitis mouse model, mimicking human Crohn’s disease, AQP8 expression is downregulated as inflammation and injury increases [255], a fact that may be better interpreted in terms of its peroxiporin activity. AQP8 was also markedly lower in inflamed colonic biopsies when compared with normal counterparts [256]. Nevertheless, other studies report an upregulation of its expression in samples from Crohn’s disease and ulcerative colitis patients. This contraposition may be explained by an adaptively triggered protein modulation in early stages of disease and later compensatory mechanisms [257].

AQP3 is highly expressed in both the proximal and distal colon, but great orders of magnitude more abundant in the latter [258]. Owing to its localization in the mucosal epithelial cells [259], a role in water transport for the formation of intestinal contents and feces was deduced. However, analogously to AQP8, downregulation of AQP3 levels was observed in the colon of Crohn’s disease and ulcerative colitis patients [257] and in drug-induced colitis as the signs of intestinal inflammation and injury progress [260]. After small bowel resection and improvement of intestinal functions in IBD rats, AQP3 was upregulated during the adaptation [259], indicating a functional significance of its levels in the pathogenesis of IBD [255,258]. In addition, AQP3 has been shown to be crucial for enterocyte proliferation [261], a process induced by the Wnt/β-catenin pathway and mediated by NOX1, as described above: AQP3 knock-out mice showed impaired capacity of proliferation in experimental models of colitis, even leading to significantly reduced mice survival. As intestinal barrier integrity was impaired on these models and improved by oral glycerol administration, glycerol-transporting ability of AQP3 was judged to be responsible for the effects observed [261]. Notwithstanding, AQP3 deletion induced a dramatic increase in *Escherichia coli* C25 translocation in AQP3 null mice [262], suggesting that production of ROS that represses bacteria replication is diminished, also leaving space to re-interpret data in light of a role of AQP3 as a peroxiporin.

Paradoxically, the same studies that firmly demonstrate a connection between NOX enzymes and IBD, supported by abundant patient data and cell-based assays, suggest that the functional link does not involve exacerbation of inflammation, but rather a decline in ROS production [263]. This would perfectly fit in a scenario in which the principal peroxiporins are downregulated, as presented above. In these circumstances, it will be really interesting to assess whether inhibition of the expression of these AQPs is induced by susceptibility genes to IBD or NOX-related cascades.

### 5.3. Neurodegenerative Diseases, NADPH Oxidases, and Peroxiporins

The lack of connective tissue in the brain incites cells to be much closer to each other than cells in peripheral tissues. Therefore, extracellular generation of ROS by NOX in the central nervous system (CNS) must be tightly controlled to avoid unwanted effects on adjacent cells in such a sensitive area. Remarkably, together with aberrant protein aggregation, major cellular symptoms of neurodegenerative diseases are oxidative stress and membrane lipid peroxidation, suggesting the cooperation of unregulated ROS fluxes near the plasma membrane barrier on establishing or aggravating these processes.

Transcripts for NOX1, NOX2, and NOX4 are present in total brains [264], but there is no detailed characterization of the expression of NOX isoforms in each neuronal subtype. Exceptions would be the recorded presence of NOX1 in dopaminergic neurons [265], NOX2 expression in CA1 hippocampal neurons [266,267] and in pyramidal neurons of socially isolated rats [268], and NOX4 on dorsal root ganglion neurons [269], in basal ganglion, and cortical neurons after stroke [270]. With respect to other cellular systems of the CNS, microglial cells are constitutively equipped with low levels of NOX2 that substantially increase upon activation [271] in agreement with their phagocytic functions; microglia also express NOX1 [272] and NOX4 [273]. Instead, astrocytes express mostly NOX4, while oligodendrocytes are the only CNS cells in which there is no data on NOX expression [274]. The precise subcellular distribution of NOX enzymes in neuron membranes is still relatively obscure. However, there is evidence for localization of NOX2 at postsynaptic terminals [275,276] in accordance with its proposed role as mediator of long-term potentiation of memory upon activation of NMDA-receptors [277,278]. Regarding AQPs, expression of all peroxiporins [279,280,281,282], including the suspected H_2_O_2_ ER transporter AQP11 [283,284], has been detected at distinct brain sites. Interestingly, all of them are upregulated under stress conditions, which goes in hand with high ROS levels, such as hypoxia, ischemia, or tumorigenesis [279,280,281,285,286]. However, the primary substrate specificity of each AQP in the brain is a matter that has not yet been investigated.

Remarkably, early research on the knock-out mice for NOX2 showed that, besides the known CGD phenotype, mice suffered from impaired memory and a synaptic deficit [287]. Though in human patients only a mild decrease in cognitive function has been described [288], a number of studies concentrated on revealing possible connections of NOX2 dysfunction with human diseases manifesting degenerative cognitive defects. A formal link between microglial NOX2 and several inflammatory brain pathologies, such as Alzheimer’s disease (AD), Parkinson’s disease (PD), and amyotrophic lateral sclerosis (ALS), was achieved [264,289]. For example, strong evidence indicates that in AD, neuron-secreted β-amyloid peptide fragments promote the assembly of NOX2 complexes in the plasma membrane of glia cells [290,291] and on neurons themselves [292,293]. The resulting increase of NOX2 activity causes alterations in the synaptic plasticity capacity of the latter, eventually leading to cell death [294]. Accordingly, in a mouse model with increased β-amyloid peptide accumulation, NOX2 deficiency improves the outcome of AD: neuronal oxidative stress and behavioral deficits strongly decrease even if β-amyloid deposits still persist, indicating that microglial NOX2 is not the cause of AD but a key amplifier of its deleterious effects [295,296]. Importantly, an unidentified source of ROS has been also shown to induce protein tau hyperphosphorylation in neurons, the second main hallmark of AD, a fact that surely warrants further investigation [297]. In PD, mitochondria are considered the primary source of oxidative stress [298]. Nevertheless, NOX proteins seem to contribute as well to the overall increase in ROS levels, as indicated by several reports using toxin-induced PD animal models and in vitro cell cultures [299,300,301,302]. As in the case of AD, microglia-mediated loss of dopaminergic neurons depends on NOX2 translocation to the cell surface, but glia cells are not the primary cause of the disease but part of an amplification loop that increases neurotoxicity [303,304]. In the end, expression of NOX2 is increased in microglia cells in the spinal cord of sporadic ALS patients, at both RNA and protein levels, leading to ROS production and oxidative damage [305]. NOX2 involvement has also been confirmed in different animal models, showing that enhanced expression of this protein accelerates disease progression [306], while its deficiency increases lifespan and ameliorates symptoms [305,307].

It is also possible that non-autonomous production of ROS by glia cells is accompanied by a parallel enhanced autocrine production of ROS in neurons. In that sense, a few recent reports have highlighted a possible role for NOX1 in the development of PD in a cell-autonomous way in dopaminergic neurons. Thus, silencing of NOX1 expression significantly reduced typical molecular features of PD, such as α-synuclein protein aggregation and high α-synuclein ubiquitin expression levels, in a drug-induced model of PD [308,309]. Analogously, silencing of the Rac1 subunit prevented neuronal death [265]. On the other hand, a large group of the inherited forms of ALS (about 15–20%) is caused by point mutations in the gene coding for the cytosolic copper/zinc superoxide dismutase (SOD1) [310]. Since NOX1 deficiency significantly increases lifespan in SOD1 mutant mice [307], NOX1 might fulfill a similar role in ALS pathogenesis as in PD.

Relevant intracellular pathways potentially affected by autocrine neuronal ROS signaling will include those depending on the known redox-sensitive transcription factors HIF1α, Nrf2, or NF-κB [274], while an additional role of redox regulation in CNS-specific transcriptional processes is not excluded. Besides, numerous receptors of the CNS and their downstream signaling (mostly kinases and phosphatases) are regulated by redox modulation of cysteine residues, including the NMDA [311], opioid [312], and gamma-aminobutyric acid receptors [313].

## 6. Conclusions

Although it is now becoming clear that the basis for redox signaling specificity starts with compartmentalization of producers and targets, the study of the localization of redox systems at particular plasma membrane regions has not gained much attention until recently. Notwithstanding, membrane architecture and organization of ROS-generating enzymes in distinct lipid domains has the potential to explain how efficient redox signal transmission could be achieved, while risk of damage intrinsically associated to ROS is minimized. Moreover, the notion that the plasma membrane actively acts as a platform for redox signaling by distributing, concentrating, and/or excluding redox-related proteins may provide insights to better understand the spatio-temporal regulation of ROS-dependent signaling pathways.

Being the main source of signaling-competent ROS in the area, of especial interest is the concomitant analysis of NOX/AQP expression in membrane rafts that has been outlined in the present review for the first time to our knowledge. We propose here that the correct expression and compartmentalization of this system at the plasma membrane is fundamental to adequately orchestrate responses in which ROS molecules operate and thus deserves further investigation. This would be of paramount importance to advance in the comprehension of complex oxidative-stress-related diseases in which intercellular and intracellular pathways seem to cooperate to cause or aggravate damage, such as IBD and neurodegenerative disorders.

## Figures and Tables

**Table 1:** The main tissue distribution of NOX isoforms exhibiting plasma membrane localization.

Isoform	Regulatory Subunits	Major Distribution Sites Reported	References
Nox1	p22^phox^ NOXA1 NOXO1 Rac	Vascular smooth muscle cells (VSMC), colon, endothelium, placenta, central nervous system (CNS)	[115,116,117,118,119]
Nox2	p22^phox^ p67^phox^ p47^phox^ p40^phox^ Rac	Phagocytes, ovary, central nervous system (CNS), cardiomyocytes, hepatocytes, endothelium, gastrointestinal tract, hematopoietic stem cells	[13,118,120,121,122,123]
Nox3	p22^phox^ NOXA1 NOXO1 Rac?	Inner ear	[124,125]
Nox4	p22^phox^	Ubiquitous, including: Endothelial cells, vascular smooth muscle cells (VSMC), kidney, lungs, hematopoietic stem cells, placenta, central nervous system (CNS)	[123,126]
Nox5	Ca^2+^ (as activator)	Lymphoid tissue, testis, ovary, spleen	[100,101]
DUOX1	Ca^2+^ (as activator) DUOXA1 (as maturation factor)	Respiratory epithelium, thyroid, gastrointestinal epithelia, salivary and rectal glands	[127,128,129,130,131]
DUOX2	Ca^2+^ (as activator) DUOXA2 (as maturation factor)	Respiratory epithelium, thyroid, gastrointestinal epithelia, salivary and rectal glands	[127,128,129,130,131]
